# Knowledge and practice of deep brain stimulation among pediatric neurology residents in Saudi Arabia

**DOI:** 10.25122/jml-2025-0021

**Published:** 2025-02

**Authors:** Hanin Jaber Algethami, Munirah Hamdan Alkhrisi, Sara Ayed Alanazi, Ruba Abdelmoaty

**Affiliations:** 1 Pediatric Neurology Department, National Neuroscience Institute, King Fahad Medical City, Riyadh, Saudi Arabia

**Keywords:** awareness, knowledge, DBS, neurology residents

## Abstract

Deep brain stimulation (DBS) is an established neurosurgical intervention for movement disorders, yet awareness among Saudi pediatric neurology residents remains limited. This study assessed the knowledge, attitudes, and perceived barriers to DBS among Saudi pediatric neurology trainees. A cross-sectional survey was conducted among pediatric neurology residents in Saudi Arabia. Participants completed a structured questionnaire assessing their familiarity with DBS indications, procedural knowledge, and training exposure. Descriptive and inferential statistics were applied. A total of 40 pediatric neurology residents participated, with a majority (87.5%) aged 26–30 years and 57.5% being women. While 65% recognized DBS as FDA-approved for adults, only 50% were aware of its pediatric approval. Knowledge of DBS targets was moderate (65%), but awareness of side effects (45%) and genetic factors influencing DBS outcomes (32.5%) was limited. Exposure to DBS-related activities was minimal, with 95% never attending a family discussion, 100% never witnessing a DBS surgery, and 80% never attending a DBS lecture. Higher residency years correlated with better DBS knowledge (*P* = 0.001), and prior patient referral was associated with higher scores (*P* = 0.028). Awareness and training in DBS among Saudi pediatric neurology residents are suboptimal. Integrating DBS education into residency curricula may improve competency and clinical decision-making.

## INTRODUCTION

Deep brain stimulation (DBS) is a transformative neurotherapeutic intervention that modulates abnormal neural activity by strategically placing electrodes in specific brain regions [1]. Initially developed for treatment-resistant movement disorders, DBS has expanded to address a range of neurological and psychiatric conditions, including Parkinson’s disease, essential tremor, dystonia, obsessive-compulsive disorder (OCD), and, more recently, certain pediatric conditions [2-5]. The US Food and Drug Administration (FDA) approved DBS for essential tremor in 1997, followed by Parkinson’s disease in 2002, and dystonia in 2003, affirming DBS’s efficacy in cases where traditional treatments fall short [6].

The clinical benefits of DBS are partly due to its reversibility and programmability, which allow for ongoing adjustment to meet patients’ changing therapeutic needs [7]. Compared to lesioning techniques, DBS provides a safer, more adaptable alternative, reducing the risks associated with more invasive surgical approaches. While the efficacy of DBS in adults is well documented, its application in pediatric neurology introduces unique challenges. These challenges stem from the developmental complexities of the pediatric brain, ethical considerations around long-term impacts on cognitive functions, and limited clinical data regarding pediatric outcomes [8,9].

In pediatric neurology, DBS has shown promise in treating severe, refractory movement disorders, particularly dystonia. Traditional treatments such as pharmacotherapy, physical therapy, and intrathecal baclofen pumps often provide limited relief for children with severe dystonia [10,11]. DBS targeting the globus pallidus internus (GPi) has demonstrated substantial improvements in motor function and quality of life for children with primary generalized dystonia [12,13]. These studies reinforce the value of DBS as a therapeutic option when other interventions fail [14].

However, awareness and understanding of DBS among medical residents remain inconsistent in Saudi Arabia and globally. Studies from the US, Germany, and the UK have highlighted significant gaps in residents' knowledge of DBS, particularly regarding its indications and risks. According to Saway *et al*. [15], Saudi medical students reported limited exposure to DBS, which may hinder their ability to recommend it as a treatment option. This knowledge gap among future practitioners, especially pediatric neurologists, may delay referrals and reduce access to DBS for pediatric patients with severe neurological conditions [16].

In Saudi Arabia, there is a pressing need for further research on DBS knowledge and practice among pediatric neurology residents. Despite the increasing demand for advanced neurotherapies, such as DBS, for pediatric patients, existing studies have not thoroughly assessed the knowledge and exposure of residents to this procedure. Addressing this gap is essential for equipping future practitioners with the skills to effectively incorporate DBS into clinical practice. This study aimed to evaluate the current level of knowledge and practical exposure to DBS among pediatric neurology trainees in Saudi Arabia. By identifying key educational deficiencies, the research will inform the development of targeted programs to better prepare residents for utilizing DBS as a therapeutic option for pediatric patients with severe neurological disorders.

## MATERIAL AND METHODS

This research utilized a descriptive, cross-sectional design conducted in March 2024, targeting trainee residents in pediatric neurology programs across various regions in Saudi Arabia. Participants who declined to provide informed consent were excluded to ensure ethical standards were upheld.

The primary data collection tool was a structured, closed-ended questionnaire to capture insights into participants' understanding and practical considerations of DBS. The questionnaire was designed in English and hosted on Google Forms to facilitate ease of access and broad reach across the target population. To ensure clarity and appropriateness of the questions, a pilot study was conducted, and Cronbach’s alpha test was performed to assess the questionnaire’s internal consistency and validate its reliability.

The link to the questionnaire was disseminated through various social media platforms to maximize participant engagement and reach a diverse sample of pediatric neurology residents. Participants were encouraged to complete the survey by selecting responses from predefined options, ensuring consistency and ease of analysis. The questionnaire was configured to accept only one submission per participant to maintain data integrity and prevent duplicate entries. All responses were securely stored within the primary researcher’s Google account, with access restricted to the research team to uphold confidentiality and data protection.

After data collection, responses were compiled and reviewed in Microsoft Excel 2016 to validate data accuracy and completeness. The dataset was then transferred to IBM SPSS Statistics software, version 21, for statistical analysis. Descriptive statistics, including frequency distributions and percentages, were used to summarize participants' responses. To explore potential associations between demographic characteristics and knowledge/practice variables, Chi-square tests and Fisher’s exact tests were applied as appropriate, considering sample size and distribution, with a significance level set at *P* < 0.05. This study adhered to the ethical guidelines for research involving human subjects. Participation was voluntary, and informed consent was obtained from all respondents. Data confidentiality was strictly maintained, with all identifiable information anonymized and securely stored.

## RESULTS

This study surveyed 40 pediatric neurology residents across various hospitals in Saudi Arabia, providing insights into their demographics, training stages, and clinical environments. Most participants (87.5%, *n* = 35) were aged between 26 and 30, while only 2.5% were 25 or younger. The sample predominantly comprised women (57.5%, *n* = 23), with men accounting for 42.5% (*n* = 17). Most participants were in advanced stages of residency, with the largest group in Residency Year 4 (35%, *n* = 14). Most (57.5%) were training at King Fahad Medical City in Riyadh, with the remainder distributed across other major hospitals in Saudi Arabia (Table 1).

**Table 1:** Demographic characteristics of study participants

Demographic characteristics	*n*	%
**Age (Years)**		
Less or equal to 25 years	1	0.02
Between 26- 30 years	35	0.87
Between 31-35 years	2	0.05
Between 36-40 years	2	0.05
**Gender**		
Female	23	0.57
Male	17	0.42
**Academic year in residency**		
R1	4	0.1
R2	5	0.12
R3	6	0.15
R4	14	0.35
R5	11	0.27
**Hospital of training**		
King Abdullah Specialist Children’s Hospital - Riyadh	5	0.12
King Fahad Medical City - Riyadh	23	0.57
King Fahad Specialist Hospital - Dammam	3	0.07
King Khalid University Hospital - Riyadh	3	0.07
Prince Sultan Military Medical City - Riyadh	3	0.07
The King Faisal Specialist Hospital - Riyadh	2	0.05
King Abdulaziz University Hospital - Jeddah	1	0.02
**Total**	**40**	**100.0**

**Figure 1:**
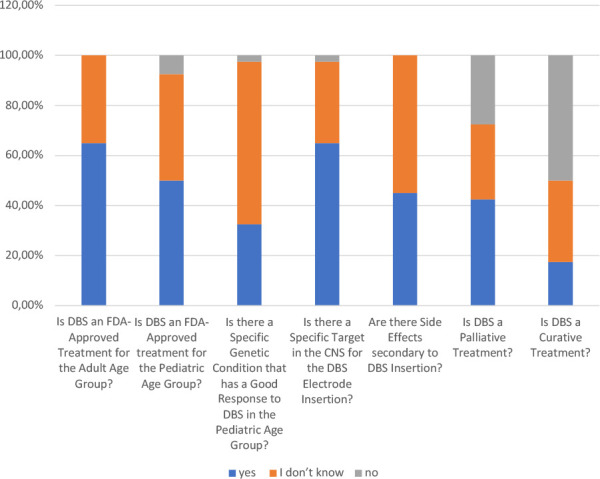
General knowledge and awareness of DBS

The study assessed participants' knowledge of DBS, yielding varied results across different aspects (Table 2, Figure 1). Awareness of DBS as an FDA-approved adult treatment was relatively high, with 65% (*n* = 26) of respondents recognizing this. However, only 50% were aware of its FDA approval for pediatric cases, indicating moderate awareness of DBS applications in younger populations. While 65% (*n* = 26) correctly identified the central nervous system targets for DBS electrode insertion, knowledge of DBS side effects was limited, with 55% (*n* = 22) unfamiliar with potential adverse outcomes. Additionally, 67.5% were unaware of genetic conditions that may respond favorably to DBS in pediatric cases. Notably, 82.5% recognized DBS as a palliative rather than a curative treatment.

**Table 2:** General knowledge of DBS

Questions	I don’t know		No		Yes	
	*n*	%	*n*	%	*n*	%
Is DBS an FDA-approved treatment for the adult age group?	14	35.0%	0	0.0%	26	65.0%
Is DBS an FDA-approved treatment for the pediatric age group?	17	42.5%	3	7.5%	20	50.0%
Does a specific genetic condition respond well to DBS in the pediatric age group?	26	65.0%	1	2.5%	13	32.5%
Is there a specific target in the CNS for the DBS electrode insertion?	13	32.5%	1	2.5%	26	65.0%
Are there side effects secondary to DBS insertion?	22	55.0%	0	0.0%	18	45.0%
Is DBS a palliative treatment?	12	30.0%	11	27.5%	17	42.5%
Is DBS a curative treatment?	13	32.5%	20	50.0%	7	17.5%

**Figure 2:**
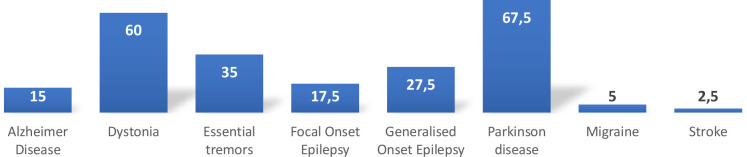
Knowledge and awareness about neurological conditions that necessitate DBS intervention in adults

**Figure 3:**
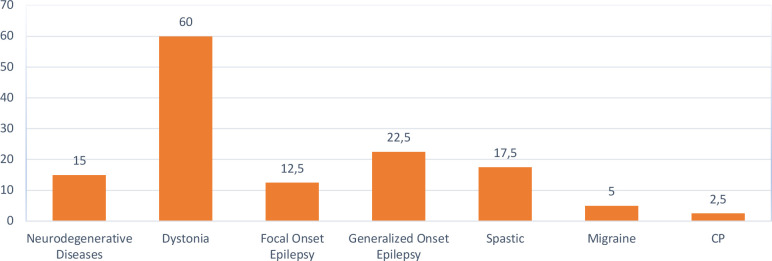
Knowledge and awareness about neurological conditions that necessitate DBS intervention in pediatrics

Regarding the conditions eligible for DBS, 67.5% of residents identified Parkinson's disease in adults, followed by dystonia (60%). In pediatric cases, dystonia (60%) was the most recognized condition for DBS, followed by generalized onset epilepsy (22.5%), which lacks FDA approval (Table 3, Figures 2 and 3).

**Table 3:** Knowledge and awareness about neurological conditions necessitating DBS intervention in adult and pediatric cases (multiple responses)

Neurological condition	*n*	%
**Adults**		
Parkinson’s disease	27	67.5%
Dystonia	24	60.0%
Essential tremors	14	35.0%
Generalized onset epilepsy	11	27.5%
Focal onset epilepsy	7	17.5%
Alzheimer’s disease	6	15.0%
Migraine	2	5.0%
Stroke	1	2.5%
**Pediatrics**		
Dystonia	24	60.0%
Generalized onset epilepsy	9	22.5%
Spasticity	7	17.5%
Neurodegenerative diseases	6	15.0%
Focal onset epilepsy	5	12.5%
Migraine	2	5.0%
Cerebral palsy (CP)	1	2.5%
All of the above	1	2.5%

**Figure 4:**
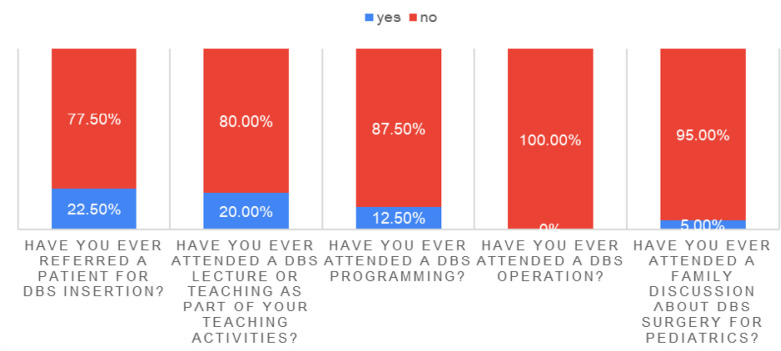
Exposure among study participants

The study also explored the practice of DBS among pediatric neurology residents. A majority had limited exposure to DBS-related activities: 95% had never attended a family discussion about DBS, 100% had never witnessed a DBS operation, and 87.5% lacked exposure to DBS programming. Educational exposure was also minimal, with only 20% attending a DBS lecture and 77.5% never referring a patient for DBS. However, 50% correctly identified the multidisciplinary team involved in DBS programming (Table 4, Figure 4).

**Table 4:** DBS practice among pediatric neurology residents

Question	Response	*n*	%
Have you ever attended a family discussion about DBS surgery for pediatrics?	No	38	95.0
	Yes	2	5.0
Have you ever attended a DBS operation?	No	40	100.0
	Yes	0	0.0
Have you ever attended DBS programming?	No	35	87.5
	Yes	5	12.5
Have you ever attended a DBS lecture or teaching as part of the academic activities?	No	32	80.0
	Yes	8	20.0
Have you ever referred a patient for DBS insertion?	No	31	77.5
	Yes	9	22.5
Who do you think is involved in the DBS programming?	Movement disorder specialist	7	17.5
	Neurosurgery	2	5.0
	Movement disorder specialist and neurosurgery	7	17.5
	Movement disorder specialist, neurosurgery, and nurse practitioner	2	5.0
	Movement disorder specialist and nurse practitioner	2	5.0
	All of the above	20	50.0

Finally, we examined the relationship between residents’ knowledge of DBS and their demographics and exposure to DBS-related activities. Knowledge scores increased significantly with the academic year (*P* = 0.001), with residents in R4 and R5 scoring the highest. No significant difference was observed based on sex (*P* = 0.12) or age (*P* = 0.09) (Table 5). While attendance at DBS-related activities did not reach statistical significance, a positive correlation was found between knowledge scores and specific DBS experiences, such as having referred a patient for DBS (*P* = 0.028) (Table 6).

**Table 5:** Knowledge score according to demographics of residents

Demographic variable	Mean knowledge score	Std. Deviation	*P* value
**Sex**			
Female	3.65	1.46	0.12
Male	2.83	1.72	
Age			
≤30	3.03	1.66	0.09
>30	4.5	0.58	
**Academic year**			
R1-R3	2.13	1.88	0.001
R4-R5	3.8	1.12	

**Table 6:** Knowledge score according to DBS-related experiences

DBS experience	Response	Mean knowledge score	Std. Deviation	*P* value
Attended family discussion about DBS	No	3.11	1.66	0.248
	Yes	4.5	0.71	
Attended DBS programming	No	3.11	1.73	0.54
	Yes	3.6	0.89	
Attended DBS lecture or teaching	No	2.94	1.74	0.067
	Yes	4.13	0.64	
Referred a patient for DBS	No	2.87	1.69	0.028
	Yes	4.22	0.97	

## DISCUSSION

DBS is a well-established neurosurgical intervention primarily used for managing movement disorders such as dystonia, Parkinson's disease, and essential tremor. In recent years, its applications have expanded to include pediatric conditions such as medically refractory epilepsy and secondary dystonia, demonstrating its potential in broader neurological contexts [1,17]. Despite these advancements, awareness and accessibility of DBS remain limited in various regions. Our study sheds light on the suboptimal knowledge of DBS among pediatric neurology residents in Saudi Arabia, a finding that mirrors reports from other regions where DBS training is not routinely incorporated into residency curricula.

Almost two-thirds of residents recognized DBS as FDA-approved for adults, but only half knew its applications in pediatric populations. This knowledge gap is consistent with prior studies in Saudi Arabia and elsewhere, where medical trainees reported limited exposure to DBS in academic and clinical settings [15,16]. One study of Saudi medical students found that approximately 59.7% were aware of the FDA approval of DBS, highlighting the need for structured DBS education during residency [16]. Similarly, a survey of Virginia Tech Carilion medical students found that while 65% felt knowledgeable about DBS, 36% were unsure of its FDA status, and 10.6% associated DBS with severe adverse outcomes [18]. These findings underscore the critical need for comprehensive DBS training to better prepare future practitioners, particularly concerning its risks and regulatory status.

This study also revealed a moderate understanding among residents regarding DBS target sites. Approximately 75.9% correctly identified basal ganglia structures, such as the globus pallidus internus (GPi) and subthalamic nucleus (STN), as common targets. The use of these areas in DBS is well-documented for treating movement disorders like dystonia and Parkinson's disease [12,14]. However, other targets are well studied in the literature, including the ventral intermediate nucleus (VIM) and anterior thalamic nucleus (ANT), for treating some diseases like obsessive-compulsive disorder and intractable epilepsy, respectively [8,19]. In 2018, the FDA approved DBS targeting the ANT for refractory focal epilepsy in adults; however, this application remains off-label for pediatric epilepsy [20,21]. Additionally, some residents believe that DBS may benefit other conditions in both adult and pediatric populations that lack FDA approval, highlighting the need for improved education on the approved clinical indications of DBS.

Pediatric DBS applications have unique challenges, especially in treating primary dystonia, a disorder affecting 15 to 30 per 1,000,000 children globally, often with genetic origins [22,23]. Despite its potential, DBS is underutilized in pediatric neurology, partly due to ethical concerns regarding long-term effects on brain development and neurocognitive functions [9]. Over half of the participants in our study recognized dystonia as a primary indication for DBS in children, consistent with global research indicating positive outcomes of DBS for refractory dystonia cases [24,25]. However, our findings show a lack of awareness regarding specific genetic etiologies that may predict better responses to DBS, highlighting an area for educational enhancement to aid practitioners in patient selection and counseling [26,27].

The study further reveals a significant gap in practical exposure to DBS among residents, with most participants reporting no firsthand experience in DBS-related surgeries, patient counseling, or treatment discussions. Indeed, some centers in Saudi Arabia have successfully conducted DBS on adults and pediatric patients; however, the number of cases still needs to be determined. A prior study reported that 20.1% of Saudi medical students received adequate DBS education, consistent with our findings of insufficient exposure among residents [16]. Given that practical exposure improves knowledge retention and procedural confidence, more collaboration with neurosurgery departments, including structured observation sessions, could benefit trainees significantly [28,10].

Our results align with international findings on DBS knowledge among trainees, underscoring the need for targeted educational interventions in neurology residency curricula. Systematic reviews by Marsh *et al*. [11] and Halpern *et al*. [29] emphasize the clinical effectiveness and safety of DBS in managing movement disorders, highlighting the value of DBS training for neurology practitioners. For instance, Marks *et al*. [25] demonstrated that structured training programs focused on pediatric movement disorders significantly improve trainees' knowledge of DBS. Furthermore, studies by Starnes *et al*. [30] and Pan *et al*. [31] emphasize the importance of comprehensive training regarding the palliative nature of DBS, which focuses on symptom management rather than curative outcomes. While most residents in our study recognized this distinction, fewer could articulate the nuances of the limitations of DBS. Addressing these gaps through targeted education could enhance trainees' competency in managing complex DBS cases.

Additionally, awareness of the side effects associated with DBS was limited among participants, with only 45% recognizing potential risks such as infection, hardware complications, and neuropsychiatric changes. This lack of awareness is concerning, as understanding these risks is crucial when counseling families about DBS [4,30].

Knowledge scores in this study were significantly associated with advanced residency levels, corroborating findings from German studies that showed students’ DBS knowledge improved with clinical exposure over time [18]. Knowledge about DBS increased with the clinical stage, confirming that experiential learning is crucial for developing competence in complex neurotherapeutic procedures [32]. This study suggests that residents in their fourth or fifth year of training had a notably higher mean knowledge score, affirming the importance of longitudinal, progressive education [5,15].

To improve DBS training among pediatric neurology residents, several strategies can be considered:

- Incorporating structured DBS training into residency curricula, including case-based learning and simulation sessions.
- Facilitating neurosurgery collaborations to provide residents with observation opportunities for DBS procedures.
- Developing mentorship programs where experienced neurologists guide junior residents in DBS case discussions and patient referrals.

By implementing these strategies, we can ensure that future pediatric neurologists are well-equipped to integrate DBS into their clinical practice, ultimately improving access to advanced neurotherapeutic options for pediatric patients in Saudi Arabia.

Despite these insights, several limitations should be considered when interpreting the results. First, the cross-sectional design of the study limits our ability to establish causal relationships, and the small sample size may reduce the generalizability of the findings. Additionally, self-reported knowledge and experience are prone to recall bias, which could influence the accuracy of the data. Future research would benefit from a longitudinal design, examining changes in DBS knowledge over time and larger, multi-center cohorts to capture a more comprehensive understanding of DBS knowledge and practice among neurology residents in diverse settings.

## CONCLUSION

This study revealed substantial knowledge and exposure gaps regarding DBS among pediatric neurology residents in Saudi Arabia, particularly in understanding its pediatric applications and procedural intricacies. The findings suggest a need for enhanced DBS education, including structured curricula and practical training, to bridge these gaps. Increased DBS knowledge was associated with advanced residency levels, supporting the importance of clinical exposure. Addressing these educational needs could better prepare residents for handling complex DBS cases, ultimately improving pediatric neurological care in the region.

## Data Availability

The data presented in this study are available upon request from the corresponding author. The data are not publicly available due to ethical restrictions.

## Personal thanks

We would like to thank Dr. Mohammad Shibly Khan, a Community Medicine specialist at King Salman Hospital, for reviewing the paper.
